# History of Minor Consent Laws for Mental Health Treatment in the US

**DOI:** 10.1001/jamahealthforum.2026.0927

**Published:** 2026-04-24

**Authors:** Hannah L. Brown, Kristen Underhill, Jaimie L. Gradus, Kimberly M. Nelson

**Affiliations:** 1 Department of Psychiatry, Boston Medical Center, Boston, Massachusetts; 2 Cornell Law School, Ithaca, New York; 3 Department of Population Health Sciences, Weill Cornell Medical College, New York, New York; 4 Department of Epidemiology, Boston University School of Public Health, Boston, Massachusetts; 5 Department of Psychiatry, Boston University Chobanian and Avedisian School of Medicine, Boston, Massachusetts; 6 Department of Community Health Sciences, Boston University School of Public Health, Boston, Massachusetts

## Abstract

**Importance:**

To facilitate access to mental health treatment for minors who are unwilling to involve parents or guardians in their care, states have enacted statutes granting minors the legal capacity to independently consent (ie, without parental/guardian consent) to mental health services. Understanding changes in these laws over time may facilitate research that assesses whether these laws increase access to care, and support legal and clinical interventions to advance minors’ access to mental health care.

**Objective:**

To analyze the adoption and amendment of minor consent laws for mental health services in all 50 states and Washington, DC, from 1950 to 2024 while focusing on the capacity of minors to independently consent and the confidentiality protections that apply to the mental health information of independently consenting minors.

**Design and Setting:**

This study used legal epidemiological methods to code state statutes, state regulations, and relevant case law regulating the legal capacity of minors in all 50 US states and Washington, DC, from 1950 to 2024 to consent independently to (1) general health care (inclusive of mental health care), (2) outpatient talk therapy, (3) mental health medications, and (4) residential treatment.

**Main Outcomes and Measures:**

The main outcome was the youngest age of capacity to consent to general health care, outpatient therapy, mental health medications, and residential treatment, respectively, as well as confidentiality protections that prohibit, enable, or require disclosure of minors’ mental health treatment information to parents or guardians.

**Results:**

From the 1950s to 2019, there was steady growth in the number of states that allowed minors to consent independently to mental health treatment, with outpatient therapy being the most common. In 2024, for the first time since states started allowing minor consent, multiple states revoked the capacity for minors to consent independently to mental health treatment. Few laws protect minors’ mental health treatment records against disclosure to their parents or guardians.

**Conclusions and Relevance:**

The results of this study suggest that research that assesses the effect of these laws (and their revocation) on clinical practice, access to care, and mental health outcomes for minors is needed to inform practice and policies that increase access to mental health care for minors in the US.

## Key Points

**Question:**

How have minor consent laws for mental health care in the US changed from 1950 to 2024?

**Findings:**

In this legal epidemiological study of all 50 states and Washington, DC, from 1950 to 2019, an increasing number of states enacted laws that allowed minors to consent to mental health care without parent/guardian involvement. More recently, several states have reversed this trend and revoked the capacity for minors to independently consent.

**Meaning:**

The study results suggest that research that assesses the effects of these laws (and their revocation) on clinical practice, access to care, and mental health outcomes for minors is needed to inform practice and policies that increase access to mental health care for minors in the US.

## Teaser

This quality improvement study examines laws in the US regarding a minor’s ability to consent to mental health care.

## Introduction

Adolescents are more likely to seek health care for sensitive health issues, including mental health concerns, if they can provide their own consent, choose the level of parental involvement in their care, and be confident that their health information will be kept private.^1,2,3,4,5,6,7,8,9,10,11,12^ To facilitate access to mental health treatment for youth who desire mental health treatment and are unwilling to involve parents in their care, some states have enacted statutes that grant minors the legal capacity to consent independently to mental health services without their parents’ involvement. However, applying these laws can require value judgments (eg, verifying whether the circumstances allow minors to consent), parsing multiple laws that may apply simultaneously (eg, consent to general health care or mental health care), and distinguishing between different types of treatment that have different legal rules (eg, the capacity to consent to outpatient treatment, medication, or residential treatment). These laws can be subtle and are subject to frequent amendments, making them challenging for clinicians, minors, and parents to understand and rely on.^13,14^

Prior reviews describing these minor consent laws have aggregated the laws without describing differences or exceptions,^7,13^ omitted administrative regulations and judicial decisions that might have interpreted state statutes,^14^ or were conducted before recent legal changes that are starting to rescind these laws.^14^ Prior reviews also reported the laws at a single point rather than exploring longitudinal changes. Using rigorous legal epidemiological methods, we built on prior legal analyses by coding and analyzing minor consent laws for mental health services and associated confidentiality protections in all 50 US states and Washington, DC, from 1950 to 2024. This analysis may assist legal, policy, and public health researchers who can harness state-level variation over time to study the effect of minor consent laws on mental health service use and outcomes among US minors. It may also help identify states where additional legal protections may be necessary. Ultimately, our goal was to clarify these complex laws for clinicians, researchers, minors, parents/guardians, policymakers, and human rights advocates and facilitate research, practice, and policies that seek to increase access to mental health care for minors in the US.

## Methods

Using best practices for creating longitudinal public health law datasets,^15,16,17^ our team followed a replicable process of identifying laws, double coding, reconciling coding differences, and analyzing relevant laws. We sought to generate a legal survey of state statutes (enacted by legislatures), state regulations (enacted by state agencies), and state and federal case law (judges’ decisions) regulating the legal capacity of minors in each state and Washington, DC, to consent independently to mental health treatment without the consent or involvement of their legal guardians (including parents or other legal guardians recognized under state law). We identified all laws using prespecified search strings in Westlaw (Thomson Reuters), conducting a search separately in each state’s statutory database and regulatory database. When we identified a relevant state law or regulation, we tracked the legal rule backward through all available past versions, examining substantive changes since its enactment for any of our variables of interest. We also reviewed all judicial decisions that cited a relevant law. We did not review laws associated with refusal of care, as those laws are distinct from minor consent laws, which are meant to increase assess for minors who would not seek desired services if parental consent was required and do not address nor remove a minor’s ability to refuse care.

We considered 2 types of law. One type of law, which we call *consent to general health care*, allows minors to consent to all forms of medical intervention. For example, Idaho Code Ann. § 39-4503 specifies, “Any person . . . who comprehends the need for, the nature of, and the significant risks ordinarily inherent in any contemplated health care services is competent to consent thereto on his or her own behalf.”^18^ We included this type of law because categories like “any contemplated health care services” include mental health care. The second type of law is specifically written to enable consent to some or all mental health care services. We coded these laws based on whether they allowed minors to independently consent to (1) outpatient talk therapy, (2) mental health medications, and (3) residential treatment.

When minor consent laws also addressed the confidentiality of minors’ health care information against disclosure to parents, we coded these confidentiality provisions, along with any protections against the disclosure of health care payer communications (eg, explanations of benefits) to parents. Because we defined minors as people younger than a state’s age of majority, which can range up to 21 years, we coded state laws that established the age of majority. We also identified conditions that states require to be met before minors can exercise the legal capacity to consent to treatment (eg, physician judgment that treatment is necessary to preserve the life or health of the patient).

We made several assumptions in coding. When there was no statute or regulation that explicitly gives minors the capacity to consent independently, we designated the age of consent as the age of legal majority in that state. When minors had the capacity to consent to general health care without limitations, we reported these laws separately; however, we interpreted these laws as encompassing all forms of mental health care unless the laws contained specific language that excluded mental health care from their scope. When minors had the capacity to consent to mental health services or treatment without limitations, we considered this to include outpatient talk therapy, medication, and residential treatment. When minors had the capacity to consent to outpatient services or outpatient treatment without limitations, we considered this to include medication. When states only allowed minors to consent to a consultation, diagnosis, or care explicitly designated as emergency care, we coded these states as lacking the capacity for minors to consent independently to mental health treatment. Further details about the study methods can be found in the eMethods in Supplement 1. Specific statutory citations are available on request. As this work did not involve human participants, it was determined by the authorship team that it did not require institutional review board review.

## Results

As illustrated in the Figure, minor consent laws encompassing general health care and laws tailored to mental health care followed similar but distinct patterns of growth and, more recently, retraction. None of the minor consent laws that applied to general health care contained language that excluded mental health care from their scope.

**Figure.**
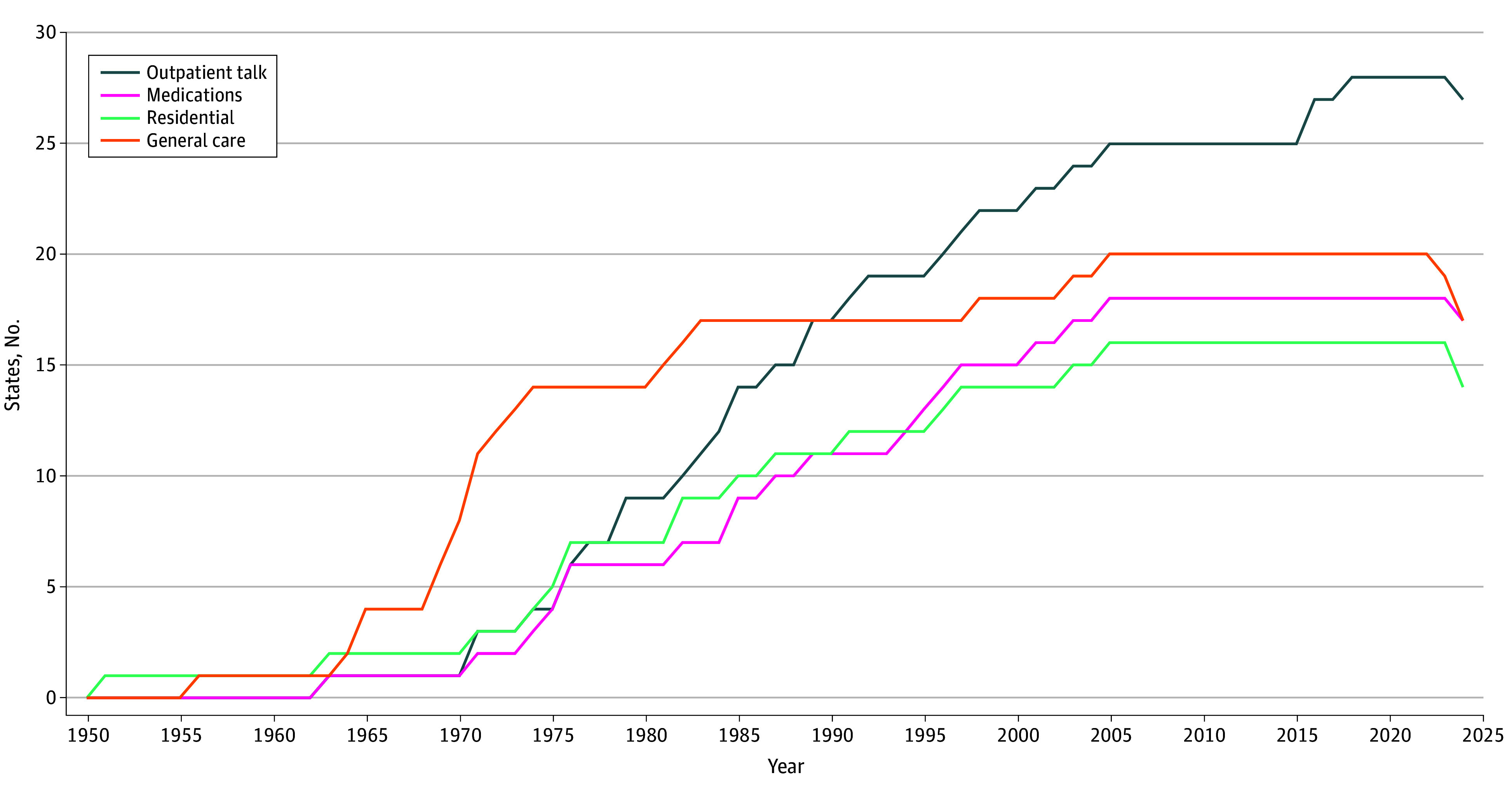
Line Graph of the Number of States With Enacted Minor Consent Laws That Enable Minors to Independently Consent to General Health Care and Mental Health Treatment

States adopted minor consent laws in similar patterns regardless of whether the scope of the law extended to health care generally or only to mental health care, and we reported both of these trends (Table 1; Figure). For each state, Table 1 reports the youngest age at which a person is legally able to consent to general health care (under a general health care law) and specific types of mental health services (under laws specific to mental health care). Table 2 reports confidentiality protections embedded in these laws.

**Table 1.** Age of Legal Capacity of Minors to Consent Independently to Care According to State Laws Applying to General Medical Care and State Laws Applying to Mental Health Care From 1950 to 2024^a^^,^^b^

State	Year	Age of majority, y	Youngest age of legal capacity to consent, y			
			General care	Outpatient talk	Medications	Residential
Alabama	1950-1971	21	21	21	21	21
	1971-1975	21	14	21	21	21
	1975-2024	19	14	19	19	19
Alaska	1950-1959	21	21	21	21	21
	1959-1977	19	19	19	19	19
	1977-2024	18	18	18	18	18
Arizona	1950-1972	21	21	21	21	21
	1972-2024	18	18	18	18	18
Arkansas	1950-1973	18^c^ or 21	18^c^ or 21	18^c^ or 21	18^c^ or 21	18^c^ or 21
	1973-1975	18^c^ or 21	0	18^c^ or 21	18^c^ or 21	18^c^ or 21
	1975-2003	18	0	18	18	18
	2003-2024	18	0	0	0	0
California	1950-1972	21	21	21	21	21
	1972-1979	18	18	18	18	18
	1979-2011	18	18	12^d^	18	18
	2011-2024	18	18	12	18	18
Colorado	1950-1963	18	18	18	18	18
	1963-2019	18	18	15	15	15
	2019-2024	18	18	12	15	15
Connecticut	1950-1992	18	18	18	18	18
	1992-2024	18	18	0^e^^,^^f^^,^^d^^,^^g^^,^^h^ or 18	18	18
Delaware	1950-1964	21	21	21	21	21
	1964-1972	21	0^d^^,^^i^ or 21	21	21	21
	1972-2024	18	0^d^^,^^i^ or 18	18	18	18
District of Columbia	1950-1974	18	18	18	18	18
	1974-2001	18	0^d^^,^^i^ or 18	0	0	0
	2001-2024	18	0^d^^,^^i^ or 18	0^j^ or 18	16^k^ or 18	18
Florida	1950-1973	21	21	21	21	21
	1973-1991	18	18	18	18	18
	1991-2024	18	18	13	18	18
Georgia	1950-2024	18	18	18	18	18
Hawaii	1950-1972	20	20	20	20	20
	1972-2016	18	18	18	18	18
	2016-2024	18	18	14	18	18
Idaho	1950-1951	21	21	21	21	21
	1951-1972	21	21	21	21	14
	1972-2005	18	18	18	18	14
	2005-2024	18	0	18	18	14
	2024	18	18	18	18	18
Illinois	1950-1969	18^c^ or 21	18^c^ or 21	18^c^ or 21	18^c^ or 21	18^c^ or 21
	1969-1971	18^c^ or 21	18	18^c^ or 21	18^c^ or 21	18^c^ or 21
	1971 to January 1979	18	18	18	18	18
	January 1979 to September 1979	18	18	14	18	18
	September 1979 to 2018	18	18	12^k^	18	18
	2018-2024	18	18	12^d^^,^^l^^,^^m^	18	18
Indiana	1950-1973	21	21	21	21	21
	1973-2024	18	18	18	18	18
Iowa	1950-2024	18	18	18	18	18
Kansas	1950-1969	18	18	18	18	18
	1969-1996	18	16^n^ or 18	18	18	18
	1996-2024	18	16^n^ or 18	14^o^ or 18	14^o^ or 18	14^o^ or 18
Kentucky	1950-1970	18	18	18	18	18
	1970-1998	18	0^e^^,^^d^ or 18	18	18	18
	1998-2024	18	0^e^^,^^d^ or 18	16	18	18
Louisiana	1950-1972	21	21	21	21	21
	1972-2024	18	0	18	18	18
Maine	1950-1969	21	21	21	21	21
	1969-1972	20	20	20	20	20
	1972-1997	18	18	18	18	18
	1997-2024	18	18	0	0	0
Maryland	1950-1973	21	21	21	21	21
	1973-1982	18	18	18	18	18
	1982-2021	18	0^d^ or 18	16	16	16
	2021-2024	18	0^d^ or 18	12	16	12
Massachusetts	1950-1987	18	18	18	18	18
	1987-2024	18	18	16	16	16
Michigan	1950-1984	18	18	18	18	18
	1984-2024	18	18	14^l^ or 18	18	18
Minnesota	1950-1971	21	21	21	21	21
	1971-1973	21	0^d^ or 21	21	21	21
	1973-1982	18	0^d^ or 18	18	18	18
	1982-2024	18	0^d^ or 18	18	18	16
Mississippi	1950-1998	21	21	21	21	21
	1998-2024	21	18	21	21	21
Missouri	1950-2024	18	18	18	18	18
Montana	1950-1969	18	18	18	18	18
	1969-1971	18	0^p^ or 18	18	18	18
	1971-1975	18	0^p^ or 18	0^q^ or 16	18	18
	1975-2023	18	0^r^ or 18	0^q^ or 16	16	16
	2023-2024	18	18	16	16	16
Nebraska	1950-1969	21	21	21	21	21
	1969-1972	20	20	20	20	20
	1972-1976	19	19	19	19	19
	1976-2018	19	19	0^s^ or 19	0^s^ or 19	0^s^ or 19
	2018-2024	19	19	0^s^ or 18	0^s^ or 18	0^s^ or 18
Nevada	1950-1965	18^c^ or 21	18^c^ or 21	18^c^ or 21	18^c^ or 21	18^c^ or 21
	1965-1973	18^c^ or 21	0^d^ or 18	18^c^ or 21	18^c^ or 21	18^c^ or 21
	1973-2024	18	0^d^ or 18	18	18	18
New Hampshire	1950-1973	21	21	21	21	21
	1973-2024	18	18	18	18	18
New Jersey	1950-1973	21	21	21	21	21
	1973-2016	18	18	18	18	18
	2016-2024	18	18	16	18	18
New Mexico	1950-1971	21	21	21	21	21
	1971-1977	18	18	18	18	18
	1977-1995	18	18	0	18	18
	1995-2007	18	18	0	14	18
	2007-2024	18	18	14	14	18
New York	1950-1983	18	18	18	18	18
	1983-1994	18	18	0	18	18
	1994-2024	18	18	0	16	18
North Carolina	1950-1965	21	21	21	21	21
	1965-1971	21	0^d^^,^^t^ or 21	18	18	18
	1971-2024	18	0^d^^,^^t^ or 18	0	0	0
North Dakota	1950-1971	18^c^ or 21	18^c^ or 21	18^c^ or 21	18^c^ or 21	18^c^ or 21
	1971-2024	18	18	18	18	18
Ohio	1950-1974	21	21	21	21	21
	1974-1989	18	18	18	18	18
	1989-2024	18	18	14^l^ or 18	18	18
Oklahoma	1950-1972	18^c^ or 21	18^c^ or 21	18^c^ or 21	18^c^ or 21	18^c^ or 21
	1972-2005	18	18	18	18	18
	2005-2024	18	18	16	16	16
Oregon	1950-1971	21	21	21	21	21
	1971-1973	21	15	21	21	21
	1973-1985	18	15	18	18	18
	1985-2024	18	15	14	14	18
Pennsylvania	1950-1970	21	21	21	21	21
	1970-1976	21	0^e^^,^^d^ or 18	21	21	21
	1976-2024	21	0^e^^,^^d^ or 18	14	14	14
Rhode Island	1950-1956	21	21	21	21	21
	1956-1972	21	16	21	21	21
	1972-2024	18	16	18	18	18
South Carolina	1950-1981	18	18	18	18	18
	1981-1991	18	0^u^ or 16	18	18	18
	1991-2024	18	0^u^ or 16	18	18	16
South Dakota	1950-1983	18	18	18	18	18
	1983-2024	18	0^e^^,^^d^ or 18	18	18	18
Tennessee	1950-2001	18	18	18	18	18
	2001-2003	18	18	16	16	16
	2003-2024	18	14	16	16	16
	2024	18	18	18	18	18
Texas	1950-2024	18	18	18	18	18
Utah	1950-2024	18	18	18	18	18
Vermont	1950-2018	18	18	18	18	18
	2018-2024	18	18	0	18	18
Virginia	1950-1989	18	18	18	18	18
	1989-2024	18	18	0	0	18
Washington	1950-1985	18	18	18	18	18
	1985-1995	18	18	14	14	14
	1995-2024	18	18	13	13	13
West Virginia	1950-2024	18	18	18	18	18
Wisconsin	1950-2024	18	18	18	18	18
Wyoming	1950-1993	19	19	19	19	19
	1993-2024	18	18	18	18	18

^a^ The 0 indicates that there is no lower age limit and all mentally competent minors can legally consent to care. Many states have laws that allow minors to consent to mental health care or specific types of mental health care, as well as laws that allow minors to consent to medical care generally. We reported lower-bound ages of capacity to consent under each of these types of laws. Laws allowing minors to consent to all medical care generally encompass mental health care as well, but because the laws are enacted separately, we reported them separately here.

^b^ The following conditions must be met for minors to be legally able to consent to care. When conditions only apply to minors of certain ages, this is noted in the table by presenting 2 ages of legal capacity to consent: 1 with conditions and 1 without. Where 2 superscript letters are presented together, both conditions must be true for minors to consent to care. Where 2 superscript letters are presented together but separated by a slash, 1 of these conditions must be true for minors to consent to care.

^c^ Minor must be female.

^d^ The failure to provide such treatment would be seriously detrimental to the minor’s well-being; the minor is a danger to themselves or others; threat to health, life, mental health, welfare, or unduly prolonging hardship.

^e^ Requiring the consent or notification of a parent or guardian would cause the minor to reject such treatment.

^f^ The provision of such treatment is clinically indicated.

^g^ The minor has knowingly and voluntarily sought such treatment.

^h^ In the opinion of the clinician providing treatment, the minor is mature enough to participate in treatment productively.

^i^ The clinician must make reasonable efforts to get parental consent.

^j^ The clinician must make a new determination every 90 days that it is still clinically indicated for the minor’s well-being to get treatment without parental consent and must document the determination.

^k^ Applies to inpatient treatment only.

^l^ Limitations on sessions or duration.

^m^ Redeterminations every 60 days until patient is age 17 years.

^n^ A parent or guardian is not reasonably available.

^o^ Head of treatment must notify parent, legal guardian, or other person known by the head of the facility to be interested in the care and welfare of the minor.

^p^ Emergency services when the only alternative is probable death or serious physical or mental damage; nonemergency services for conditions that will endanger health or life if services would be delayed by obtaining consent from a parent or legal guardian.

^q^ Urgent counseling.

^r^ Emergency services when, in good faith and reasonable belief supported by fact, aid is the only alternative to probable death or irreparable physical damage; no capacity to consent to nonemergency.

^s^ Any public or private hospital, other treatment facility, or program for treatment of mental illness, substance dependence, or personality disorders must be a facility that is licensed to provide services for persons who have mental illness or substance dependence or both.

^t^ If parents refused consent, the clinician must consult another physician before proceeding.

^u^ Deemed necessary, except an operation, that must be essential to health or life and 2 physicians agree.

**Table 2.** State-Level Confidentiality Protections for the Mental Health Care Information From 1950 to 2024

State	Year	General care	Outpatient talk	Medications	Residential
Alabama	1950-1971	NA	NA	NA	NA
	1971-1975	None	NA	NA	NA
	1975-2024	None	NA	NA	NA
Alaska	1950-1959	NA	NA	NA	NA
	1959-1977	NA	NA	NA	NA
	1977-2024	NA	NA	NA	NA
Arizona	1950-1972	NA	NA	NA	NA
	1972-2024	NA	NA	NA	NA
Arkansas	1950-1973	NA	NA	NA	NA
	1973-1975	None	NA	NA	NA
	1975-2003	None	NA	NA	NA
	2003-2024	None	None	None	None
California	1950-1972	NA	NA	NA	NA
	1972-1979	NA	CD	NA	NA
	1979-2011	NA	CD	NA	NA
	2011-2024	NA	CD^a^	NA	NA
Colorado	1950-1963	NA	NA	NA	NA
	1963-2019	NA	CD	CD	CD
	2019-2024	NA	CD	CD	CD
Connecticut	1950-1992	NA	NA	NA	NA
	1992-2021	NA	MC	NA	NA
	2021-2024	NA	CD	NA	NA
Delaware	1950-1964	NA	NA	NA	NA
	1964-1972	None	NA	NA	NA
	1972-2024	None	NA	NA	NA
District of Columbia	1950-1974	NA	NA	NA	NA
	1974-2001	CD	CD^b^	CD^b^	CD^b^
	2001-2024	CD	CD^c^	CD^c^	NA
Florida	1950-1973	NA	NA	NA	NA
	1973-1991	NA	NA	NA	NA
	1991-2024	NA	CD	NA	NA
Georgia	1950-2024	NA	NA	NA	NA
Hawaii	1950-1972	NA	NA	NA	NA
	1972-2016	NA	NA	NA	NA
	2016-2024	NA	CD	NA	NA
Idaho	1950-1951	NA	NA	NA	None
	1951-1972	NA	NA	NA	NA
	1972-2005	NA	NA	NA	None
	2005 to July 2024	None	NA	NA	None
	July 2024 to December 2024	None	NA	NA	NA
Illinois	1950-1969	NA	NA	NA	NA
	1969-1971	MC	NA	NA	NA
	1971 to January 1979	NA	NA	NA	NA
	January 1979 to September 1979	NA	CD	NA	NA
	September 1979 to 2018	NA	CD	NA	NA
	2018-2024	NA	CD	NA	NA
Indiana	1950-1973	NA	NA	NA	NA
	1973-2024	NA	NA	NA	NA
Iowa	1950-2024	NA	NA	NA	NA
Kansas	1950-1969	NA	NA	NA	NA
	1969-1996	None	NA	NA	NA
	1996-2024	None	None	None	None
Kentucky	1950-1970	NA	NA	NA	NA
	1970-1998	None	NA	NA	NA
	1998-2024	None	None	NA	NA
Louisiana	1950-1972	NA	NA	NA	NA
	1972-2024	CD	NA	NA	NA
Maine	1950-1969	NA	NA	NA	NA
	1969-1972	NA	NA	NA	NA
	1972-1997	NA	NA	NA	NA
	1997-2024	NA	CD	CD	CD
Maryland	1950-1973	NA	NA	NA	NA
	1973-1982	NA	NA	NA	NA
	1982-2021	CD	CD	CD	CD
	2021-2024	CD	CD	CD	CD
Massachusetts	1950-1987	NA	NA	NA	NA
	1987-2024	NA	None	None	None
Michigan	1950-1984	NA	NA	NA	NA
	1984-2024	NA	CD^d^	NA	NA
Minnesota	1950-1971	NA	NA	NA	NA
	1971-1973	CD	NA	NA	NA
	1973-1982	CD	NA	NA	NA
	1982-2024	CD	NA	NA	None
Mississippi	1950-1998	NA	NA	NA	NA
	1998-2024	None	NA	NA	NA
Missouri	1950-2024	NA	NA	NA	NA
Montana	1950-1969	NA	NA	NA	NA
	1969-1971	None	NA	NA	NA
	1971-1975	None	None	NA	NA
	1975-2023	None	None	None	None
	2023-2024	NA	None	None	None
Nebraska	1950-1921	NA	NA	NA	NA
	1921-1969	NA	NA	NA	NA
	1969-1972	NA	NA	NA	NA
	1972-1976	NA	NA	NA	NA
	1976-2018	NA	None	None	None
	2018-2024	NA	None	None	None
Nevada	1950-1965	NA	NA	NA	NA
	1965-1973	None	NA	NA	NA
	1973-2024	None	NA	NA	NA
New Hampshire	1950-1973	NA	NA	NA	NA
	1973-2024	NA	NA	NA	NA
New Jersey	1950-1973	NA	NA	NA	NA
	1973-2016	NA	NA	NA	NA
	2016-2024	NA	CD	NA	NA
New Mexico	1950-1971	NA	NA	NA	NA
	1971-1977	NA	NA	NA	NA
	1977-1995	NA	None	NA	NA
	1995-2007	NA	None	None	NA
	2007-2024	NA	None	None	NA
New York	1950-1983	NA	NA	NA	NA
	1983-1994	NA	None	NA	NA
	1994-2016	NA	None	None	NA
	2016-2024	NA	MC	MC	NA
North Carolina	1950-1965	NA	NA	NA	NA
	1965-1971	None	NA	NA	NA
	1971-2024	None	CD	CD	CD
North Dakota	1950-1971	NA	NA	NA	NA
	1971-2024	NA	NA	NA	NA
Ohio	1950-1974	NA	NA	NA	NA
	1974-1989	NA	NA	NA	NA
	1989-2024	NA	CD	NA	NA
Oklahoma	1950-1972	NA	NA	NA	NA
	1972-2005	NA	NA	NA	NA
	2005-2024	NA	None	None	None
Oregon	1950-1971	NA	NA	NA	NA
	1971-1973	CD	NA	NA	NA
	1973-1985	CD	NA	NA	NA
	1985-2024	CD	CD	CD	NA
Pennsylvania	1950-1970	NA	NA	NA	NA
	1970-1976	None	NA	NA	NA
	1976-2005	None	None	None	None
	2005-2024	None	MC	MC	MC
Rhode Island	1950-1956	NA	NA	NA	NA
	1956-1972	None	NA	NA	NA
	1972-2024	None	NA	NA	NA
South Carolina	1950-1981	NA	NA	NA	NA
	1981-1991	None	NA	NA	NA
	1991-2024	None	NA	NA	None
South Dakota	1950-1983	NA	NA	NA	NA
	1983-2024	None	NA	NA	NA
Tennessee	1950-2001	NA	NA	NA	NA
	2001-2003	NA	None	None	None
	2003 to July 2024	None	None	None	None
	July 2024 to December 2024	None	NA	NA	NA
Texas	1950-2024	NA	NA	NA	NA
Utah	1950-2024	NA	NA	NA	NA
Vermont	1950-2018	NA	NA	NA	NA
	2018-2024	NA	None	NA	NA
Virginia	1950-1989	NA	NA	NA	NA
	1989-2005	NA	None	None	NA
	2005-2024	NA	CD^e^	CD^e^	NA
Washington	1950-1985	NA	NA	NA	NA
	1985-1995	NA	CD	MC^f^; CD^g^	MC
	1995-2019	NA	CD	MC^f^; CD^g^	MC
	2019-2024	NA	CD	CD	CD
West Virginia	1950-2024	NA	NA	NA	NA
Wisconsin	1950-2024	NA	NA	NA	NA
Wyoming	1950-1993	NA	NA	NA	NA
	1993-2024	NA	NA	NA	NA

Abbreviations: CD, clinician discretion; MC, mandatory confidentiality; NA, minors do not have the legal capacity to independently consent to the service; none, no confidentiality protections.

^a^ After consulting with the minor.

^b^ If there are severe complications, major surgery, a failure to inform that would seriously jeopardize the safety and health of the minor, or that “to inform them would benefit the minor’s physical and mental health and family harmony.”

^c^ If there is an emergency or the patient needs to be hospitalized or protected from injury.

^d^ Can only disclose if there is a substantial threat of harm to the minor or another person.

^e^ The minor is an adult for records access, but nothing bars parents from getting health records, and a clinician can withhold if disclosure is reasonably likely to cause substantial harm to the minor or another person.

^f^ Inpatient only.

^g^ Outpatient only.

### General Health Care Laws

#### Early Adopters (1950s)

In 1956, Rhode Island became the first state to legislate that minors could independently consent to health care generally, allowing minors 16 years or older to consent to routine and emergency medical care. No confidentiality protections were included to prevent disclosure of a minors' health care information to their parents.

#### Rapid Diffusion (1960s and 1970s)

By 1970, Delaware, Illinois, Kansas, Kentucky, Montana, Nevada, and North Carolina also passed laws that allowed minors to independently consent to health care generally. Apart from Illinois, these laws did not prohibit clinicians from sharing minors’ health care information with their parents. Most of these states required clinicians to verify or fulfill certain conditions before allowing a minor to consent independently to care (eg, determining that requiring parental consent would result in the denial or delay of needed treatment).

By the end of the 1970s, Alabama, Arkansas, Washington, DC, Louisiana, Minnesota, Oregon, and Pennsylvania joined the list of states with minor consent laws for general health care. These laws included conditions like minimum age requirements or a clinician judgment that there is a threat to life or health. Alabama, Arkansas, and Pennsylvania did not address confidentiality in their laws. Washington, DC, Louisiana, Minnesota, and Oregan explicitly stated that it was a clinician’s discretion whether to disclose minors’ health care information to parents.

#### Late Adoption and Stabilization (1980s to 2010s)

Maryland, South Carolina, South Dakota, Mississippi, Idaho, and Tennessee approved legislation that allowed minors to consent independently to general health care during this time. Maryland, South Carolina, and South Dakota passed their legislation during the 1980s. In 1998, Mississippi, where the age of majority was 21 years, passed a law allowing 18-year-olds to independently consent to health care, thus meeting our definition of giving minors the capacity to consent independently. Idaho passed legislation that allowed minors to independently consent to general health care in 2005. In 2003, minors were granted the ability to independently consent to general health in Tennessee via an opinion that was issued by the state attorney general specifying that a minor 14 years or older should be presumed capable of consenting to health care independently, although that presumption can be rebutted. South Carolina, South Dakota, Idaho, and Tennessee did not include confidentiality protections in their legislation, whereas Maryland and Mississippi included language that allowed clinician discretion to determine disclosure of health care information to parents.

### Mental Health–Specific Laws

#### Early Adopters (1950s and 1960s)

In 1951, Idaho was the first state to pass a statute explicitly allowing minors to consent independently to mental health care. This law was limited in scope, giving minors 14 or older the capacity to consent independently to mental health hospitalization for a maximum of 3 days, with mandatory notification of parents. In 1963, Colorado passed a law explicitly allowing minors to consent to any kind of mental health treatment at 15 years or older. This law gave clinicians discretion to disclose minors’ health care information to parents.

#### Rapid Diffusion (1970s and 1980s)

Throughout the 1970s and 1980s, 17 states adopted minor consent laws for mental health treatment. These states used early laws as a template and began to experiment with lower age restrictions, including laws that allowed independent consent by minors as young as age 12 years (California and Illinois) or laws that did not specify a lower bound (9 states). Many of these new laws applied only to some forms of treatment or specified conditions. Colorado, Washington, DC, Maryland, Massachusetts, Montana, North Carolina, and Washington allowed minors to consent to outpatient talk therapy, mental health medications, and residential treatment with no conditions. Nebraska allowed minors to consent to all treatment modalities, but only if the treatment facility was licensed to provide services for persons with mental illness, substance dependence, or both. Illinois, New Mexico, and New York enacted laws that allowed minors to consent to outpatient talk therapy only, with no conditions. California allowed minors to consent independently to outpatient talk therapy, but only when the minor was a danger to themselves or others. Similarly, Ohio and Michigan granted minors the right to consent to outpatient talk therapy but with restrictions, such as time limits (Ohio) or the requirement that care does not undermine the parents’ values (Michigan). Oregon and Virgina lowered the age of consent for outpatient talk therapy and mental health medications, but not for residential treatment. In contrast, Minnesota allowed minors to consent independently to residential treatment, but not to medication or outpatient therapy. Only half of the laws passed during this time included language addressing confidentiality, most of which allowed clinicians discretion over disclosure to parents.

#### Differentiation, Increased Specificity, and Experimentation (1990s Through 2010s)

Throughout this period, the number of states with mental health–specific laws stabilized, but the laws themselves diversified as states’ policy preferences grew more specific. Laws set new kinds of conditions (eg, if requiring parental consent would cause the minor to reject such treatment [eg, Connecticut]), differentiated among different types of treatment (eg, different ages restrictions or conditions for outpatient vs inpatient therapy [eg, Hawaii, New Jersey, South Carolina, and Vermont]), and limited the number of sessions clinicians could have with minors without parental consent (eg, Washington, DC, and Illinois). In general, laws tended to be more expansive in the capacity of minors to consent to outpatient talk therapy (eg, New Mexico), compared with medication/residential, and some laws were enacted specifically to cover outpatient counseling while excluding other kinds of care (eg, Florida and Kentucky).

Confidentiality protections created or amended during this time were more protective compared with prior eras. Some states (eg, Connecticut, New York, Pennsylvania, and Washington) barred clinicians from disclosing information to parents. Fourteen other states left confidentiality up to a clinician’s discretion, and 13 states did not provide any confidentiality protections.

### Retractions of Minor Consent for Both Types of Law (2020s)

In 2023 and 2024, 3 states passed specific statutes that truncated or eliminated the capacity for minors to consent independently to health care. This trend included changes to laws addressing general health care, as well as changes or repeals of laws specific to mental health care.

In 2023, Montana repealed a provision that had allowed minors to consent independently to nonemergency care “for conditions that will endanger the health or life of the minor if services would be delayed” by parental consent.^19^ Instead, Montana minors can now independently consent to general health care only when they need “immediate health care” and the clinician believes “that the giving of aid is the only alternative to probable death or irreparable physical damage.” This narrow language eliminates independent access to most care. In the same bill, the legislature repealed a state law that allowed minors of any age to consent to mental health counseling under certain conditions. This choice suggests that the legislature was informed about the set of laws that would be affected by the new bill and that they exercised judgment over which laws to repeal. The legislature did not change the state law that allowed minors 16 years or older to “consent to receive mental health services.” This suggests that the state’s 2023 changes eliminated minors’ capacity to consent to general health care as well as the capacity of minors younger than 16 years to consent to mental health counseling. However, this also suggests that Montana intended for minors to retain their ability to consent independently to mental health services from age 16 years and onward. Table 1 and Figure reflect this coding.

In 2024, Tennessee enacted a law allowing minor consent only for emergency treatment; the law states that “[e]xcept as otherwise provided by statutory law, case law, or court order, a government entity, a healthcare provider, or any other person shall not knowingly take any of the following actions with regard to a minor without first obtaining the consent of a parent of the minor: (1) Treat, profess to diagnose, operate on, or prescribe for any physical ailment, physical injury, or deformity; (2) Prescribe, dispense, deliver, or administer any drug or medication; (3) Render psychological services . . . or (4) Render counseling services.”^20^

Similarly, in 2024, Idaho passed a law specifying that, with the exception of an emergency with threat of death or “imminent, irreparable physical injury,” “except as otherwise provided by court order, an individual shall not furnish a health care service or solicit to furnish a health care service to a minor child without obtaining the prior consent of the minor child’s parent.”^21^

Neither the Tennessee nor the Idaho statute repealed other statutory provisions. However, because the new restrictive laws are more recent, and conflict with laws allowing minors to consent to care, we code them in Table 1 and Figure as eliminating the capacity for minors to consent to all forms of general and mental health care.

## Discussion

State laws that enable minors to consent independently to mental health treatment may facilitate access to mental health treatment for youth who desire mental health treatment and are unwilling to involve parents in their care. These laws have evolved substantially over the past 75 years.

Before 1951, no state had legislation that allowed minors to independently consent to mental health treatment. Coinciding with the social justice and civil rights movements^22,23^ and advances in mental health care for minors (eg, approval of attention deficit/hyperactivity disorder medications for pediatric use,^24^ growth in evidence-based, nonpharmacological interventions for minors^25,26^), the 1960s and 1970s saw the first burst of states that allowed minors to consent independently to mental health treatment either via minor consent laws for general health care or laws specific to mental health treatment.

Growth in minor consent laws continued through the 1980s and 1990s. The steady increase in minor consent laws during this time may reflect an international movement to establish the rights of children, including establishing children’s rights to access high-quality health care (eg, adoption of The Convention on the Rights of the Child by the United Nations General Assembly^27^). While the ability of minors to consent independently to outpatient therapy remained the most common during this period, by the mid-1990s, more states allowed minors to consent independently to mental health medications than to residential treatment. This may be in reaction to the substantial growth in the use of pharmacological mental health interventions for minors during this time (eg, the increase in medication management for attention deficit/hyperactivity disorder^24,28^ and use of antidepressants^29^ for pediatric patients), which some consider to coincide with the start of the overmedicalization of mental health care in the US.^30,31^

During the early 2000s, the number of states that allowed minors to consent independently to mental health treatment slowly grew and then stabilized. That changed in 2023 and 2024, when several states passed laws markedly truncated minors’ ability to independently consent to general and mental health care, which is likely associated with the growing “parental rights” movement in the US.^32,33^ These reversions are not unique to minor consent laws; they can also be seen in the increasing restrictions being placed on youth access to reproductive services and gender-affirming care.^34^ As this is the first time in history that the ability for minors to independently consent to care is being revoked, it is critical that researchers assess the effect of these reversions on clinical practice, access to care, and mental health outcomes among minors. Further, clinicians and health care organizations should consider the potential that the minor consent laws in the state(s) where they practice may be revoked and respond through clinical practices and advocacy as appropriate.

In addition to the history of growth and now recission of these laws, there is substantial heterogeneity in the features of minor consent laws that may make them challenging to navigate. First, many states allow minors to independently consent to care only if certain conditions are met, such as emergencies or the likelihood that parental consent will delay care, and the burden for verifying these conditions (including the legal risks of error) rests with clinicians. Second, many states do not protect minors’ mental health information against parental disclosure. The most common arrangement is to leave disclosure of minors’ mental health treatment information up to the discretion of the clinician. This can create an additional source of uncertainty and concern for clinicians, and it can deter minors from seeking treatment or disclosing important information. Third, it is common for states to set different ages and impose different conditions for outpatient treatment, medication, and residential treatment. This poses a substantial challenge for clinicians and minors in developing integrated or flexible treatment plans. So, although many states have laws in place that allow minors to independently consent to mental health care, the complexity of these laws may serve as a barrier to reliance on their use.

### Limitations

This study had limitations. Because mental capacity to consent is already a prerequisite to providing consent, our findings do not apply to minors who lack mental competence. Our search for relevant laws included search terms associated with mental health, but not to specific treatments (eg, cognitive behavioral therapy), so laws that were codified without a reference to mental health may have been overlooked. We identified and coded confidentiality protections that were embedded in minor consent laws, but we did not run an independent search for confidentiality protections that were enacted separately. Additionally, our search was not optimized to identify laws that had been long discontinued, for which an updated or repealed version may have been unavailable.

## Conclusions

This study found that the number of states that allow minors to independently consent to mental health care has grown substantially over the past 75 years. However, there have also been recent revocations. These laws are complex, which is likely to create difficulties for clinicians who work with minors and minors who are seeking care. Further, these laws are not sufficient to guarantee access to care for minors; clinicians must also be willing to provide this care, and arrangements for payment and confidentiality are critical. This detailed, longitudinal assessment of the law may facilitate research that assesses the effect of these laws (and their revocation) on clinical practice, access to care, and mental health outcomes for minors, identify states where additional legal protections may be necessary, and inform practice and policies that increase access to mental health care for minors in the US.

## Notes

Supplement 1. eMethods. Additional details about coding procedures and definitions used for coding minor consent laws for general health care and mental health treatment in the United States

Supplement 2. Data sharing statement
