# Supplementary material for: History of Minor Consent Laws for Mental Health Treatment in the US
Source: JAMA Health Forum. 2026 Apr 24;7(4):e260927. doi: 10.1001/jamahealthforum.2026.0927 (PMC13109796; doi:10.1001/jamahealthforum.2026.0927)
Supplement: Supplement 1. — eMethods. Additional details about coding procedures and definitions used for coding minor consent laws for general health care and mental health treatment in the United States [file jamahealthforum-e260927-s001.pdf]

Supplemental Online Content

Brown HL, Underhill K, Gradus JL, Nelson KM. History of minor consent laws for mental health treatment in the US. *JAMA Health Forum*. 2026;7(4):e260927. doi:10.1001/jamahealthforum.2026.0927

eMethods. Additional details about coding procedures and definitions used for coding minor consent laws for general health care and mental health treatment in the United States

This supplemental material has been provided by the authors to give readers additional information about their work.

© 2026 Brown HL et al. *JAMA Health Forum*.

eMethods. Additional details about coding procedures and definitions used for coding minor consent laws for general health care and mental health treatment in the United States

Before identifying and coding laws that met our inclusion criteria, we created a codebook that specified and enumerated the features of state law that affect minors' capacity to consent to mental health care. Drawing on prior literature, we generated a list of legal variables of interest pertaining to state laws on minors' capacity to consent independently to mental health treatment (e.g., the youngest age at which someone can consent to outpatient counseling for mental healthcare without parental involvement, whether the law requires clinicians to verify that certain conditions are met before allowing minors to consent, etc.). We organized these variables into a coding scheme with pre-specified answers to coding questions (e.g., yes/no/silent, numerical ages). We revisited our coding scheme and codebook over the course of data collection to incorporate new variables of interest (e.g., what conditions must be met for a minor to consent independently to medication treatment?) and to refine any categorical coding choices (e.g., "mental health services," where not defined by law, encompasses outpatient care, medication, and residential care).

Four law student research assistants (RAs) implemented our search for laws and initial coding, supervised by KU. Searches and coding were initially carried out in June 2020-May 2021, and they covered the period from 1900 through 2021. RAs were assigned in pairs to each state, and each RA independently searched for and coded statutes. The RAs then met in pairs to resolve any discrepancies in independent coding, referring differences to KU and KN. We searched for relevant laws using the following search strings in each Westlaw database for state statutes, and again in each Westlaw database for state administrative regulations: (1) (minor!

© 2026 Brown HL et al. *JAMA Health Forum*.

adolesc! child! age!) /p ("medical care" "health" "healthcare" doctor! physician! provider! treat! prevent! diagnos!), and (2) (minor! adolesc! child! age!) /p (mental! behav! drug alcohol! addict! substanc!) /p ("medical care" "health" "healthcare" doctor! physician! provider! treat! prevent! diagnos!). Results of string (2) are a subset of the results from string (1), but because the default results of a Westlaw search are organized by relevance, we ran both searches as a double-check that we had identified the laws most relevant for mental health. KU and KN worked jointly to update searches. We did not conduct searches of databases of judicial case law; instead, when a law met our inclusion criteria, we read all state court cases that cited to that law to identify any changes over time due to judicial interpretation. We updated the search to cover laws in effect through December 31, 2024, and we conducted updates during September 2024-January 2025. This culminated in the review of approximately 9,085 state statutes and 9,978 administrative regulations, across all U.S. states and the District of Columbia.

Where we identified a relevant state law or regulation, we tracked the legal rule backward through all available past versions, examining substantive changes since its first effective date for any of our variables of interest. We considered a "substantive change" to be any amendment that changes the value of a variable that we included in this analysis, such as changing the age at which a minor can consent, the presence/absence of a condition required to enable minors to consent, or a change in confidentiality rules from permissive (letting clinicians decide about disclosure) to mandatory (barring all disclosures).

We were occasionally unable to obtain versions of legislation from specific past years. In these situations, we used all available contextual information to identify possible changes at the missing time point (e.g., legislative history, legislative findings, case law, editors' comments), and if we could not identify a change, we conservatively assumed that no changes had been

© 2026 Brown HL et al. *JAMA Health Forum*.

made from the time of inception to the time of the earliest available version. All relevant laws were Shepardized, a process of reviewing any citing references (e.g., cases, regulations) that may affect the validity or application of the law. Data were abstracted from legal source material into an Excel spreadsheet for each state, noting all relevant amendments and their years. HB and KU reviewed findings to identify coding errors.

For outpatient care, medication, and residential care, we identified the youngest age at which mentally competent minors have capacity to consent independently. Sometimes multiple laws provided capacity to consent to the same form of care (e.g., a state law allowing minors to consent to all medical treatment at age 16, alongside a state law allowing minors to consent to outpatient mental health counseling at age 12 if a clinician determines that involving parents would be detrimental to care). Where this occurred, we coded the youngest age at which the minor has capacity to consent both with any conditions specified by law (e.g., involving parents would be detrimental to care) and without conditions. For every amendment or new law, we recorded the effective date as the first full month in which the law was in effect. To simplify analyses for this paper, we drop the month and consider only the year in which the change took place. A 2020 date, therefore, means that the change in law took effect at some time during the calendar year 2020.

Where laws required that certain conditions be present for minors to consent independently to care, we coded these conditions when they imposed a burden on clinicians (e.g., the clinician must keep records) or when they imposed meaningful limits on access (e.g., the minor can only consent to mental health treatment at a frequency of once per week). When laws specified conditions that would apply to any person seeking treatment, not just minors (e.g., capacity to understand risks and benefits, treatment is voluntary), we did not consider these to be

© 2026 Brown HL et al. *JAMA Health Forum*.

meaningful limits on access. We coded language like "shall," "must," or "will" as mandatory, and language like "may" or "can" as permissive.

When we coded confidentiality protections, we designated the confidentiality of minors' information as "mandatory" if the law prohibits the clinician from disclosing information to parents. If the clinician has judgment about whether to disclose information to parents, we coded this as "clinician discretion." Where the law does not specifically address the disclosure of minors' mental health treatment information to parents, we coded confidentiality protections as absent.
