# Supplementary material for: History of Minor Consent Laws for Mental Health Treatment in the US
Source: JAMA Health Forum. 2026 Apr 24;7(4):e260927. doi: 10.1001/jamahealthforum.2026.0927 (PMC13109796; doi:10.1001/jamahealthforum.2026.0927)
Supplement: Supplement 2. — Data sharing statement [file jamahealthforum-e260927-s002.pdf]

Data Sharing Statement

Brown. History of Minor Consent Laws for Mental Health Treatment in the US. *JAMA Health Forum*. Published April 24, 2026. doi:10.1001/jamahealthforum.2026.0927

Data
Data available: Yes
Data types: Data (not involving human participants)
How to access data: Data is available upon request to Dr. Kimberly Nelson.
When available: With publication

Supporting Documents
Document types: None

Additional Information
Who can access the data: Researchers whose proposed use of the data has been approved
Types of analyses: For any purpose
Mechanisms of data availability: Data will be made available with investigator support after the approval of a proposal and with a signed data access agreement.
